# Supplementary material for: Evaluation of the Chemotherapy Drug Response Using Organotypic Cultures of Osteosarcoma Tumours from Mice Models and Canine Patients
Source: Cancers (Basel). 2021 Sep 29;13(19):4890. doi: 10.3390/cancers13194890 (PMC8507898; doi:10.3390/cancers13194890)
Supplement: Supplementary file 1 [file cancers-13-04890-s001.zip › cancers-1277171-supplementary.pdf]

Supplementary Material: Evaluation of the Chemotherapy Drug Response Using Organotypic Cultures of Osteosarcoma Tumours from Mice and Canine Patients

Bénédicte Brulin, John C Nolan, Tecla Marangon, Milan Kovacevic, Mathias Chatelais, Pierre Meheust, Jérome Abadie, Louis-Romée Le Nail, Philippe Rosset, Meadhbh Á Brennan, Pierre Layrolle

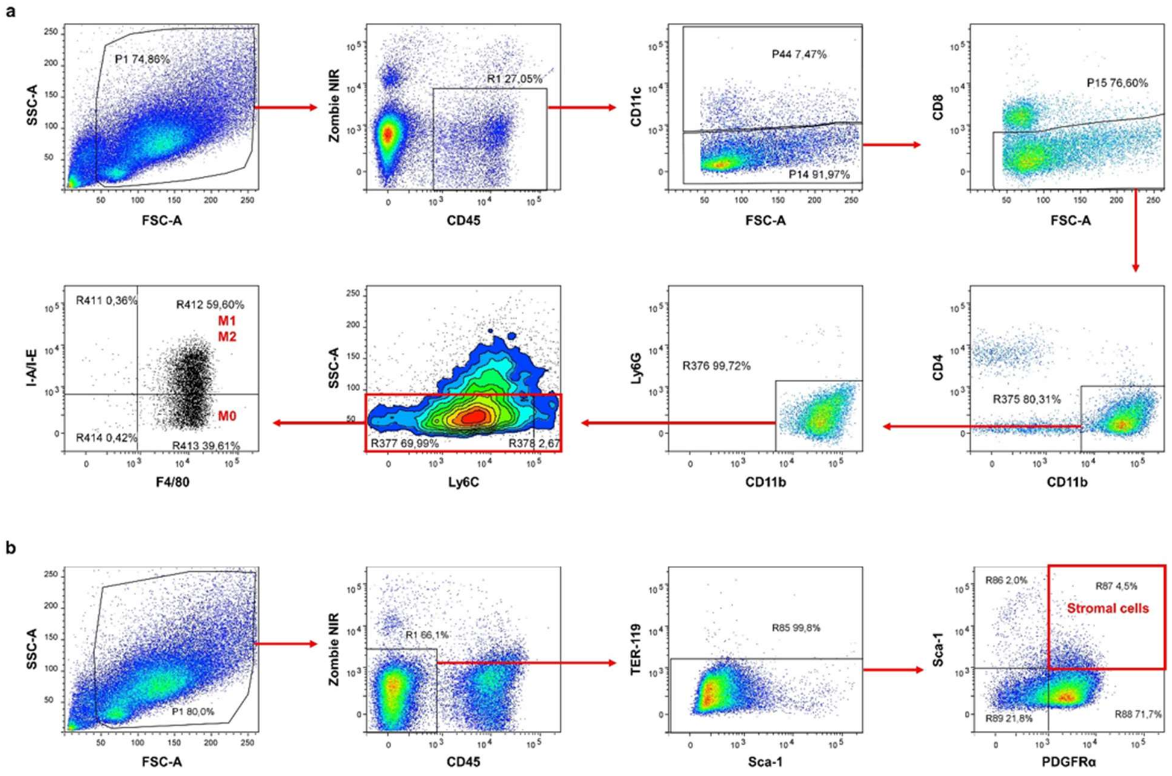

Figure S1. Flow cytometry gating of MosJ (murine osteosarcoma) tumours. (a) Gating strategy for monocytes/macrophages: viable hematopoietic cells were determined by using CD45 marker and Zombie NIR to discriminate between live and dead cells. Dendritic cells (CD11c+), T-lymphocytes (CD8+ and CD4+) and neutrophils (Ly6G+) were excluded from analysis. Then, CD11b+ cells were taken into account and divided into 2 subset: SSC-A low Ly6C low/med and SSC low Ly6C high, 2 subpopulations of macrophages and monocytes. For both Ly6C low/med and Ly6C high subpopulations, I-A/I-E marker was used with F4/80 to discriminate between M1 M2 (F4/80+ I-A/I-E+) and M0 (F4/80+ I-A/IE-) macrophages. (b) Gating strategy for stromal cells: viable hematopoietic cells were determined by using CD45 marker and Zombie NIR to discriminate between live and dead cells. CD45+ cells were excluded from analysis: only viable CD45- cells were taken into account. Then, erythroid lineage (TER119) were excluded and stromal cells were considered as Sca1+ PDGFRα+.

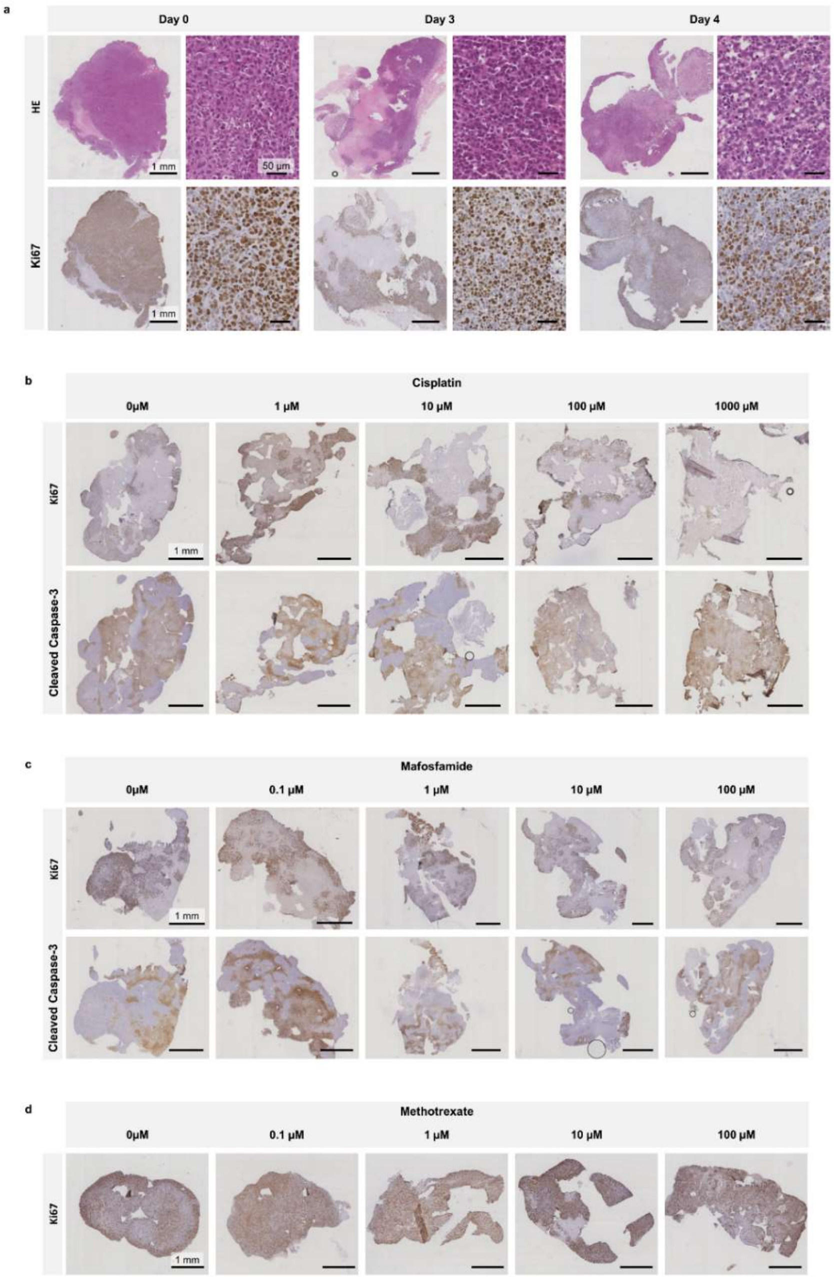

Figure S2. Organotypic slices of K-HOS (human osteosarcoma). (a) H&E and Ki67 staining of 200 µm organotypic slices at day 0, 3, and 4. (b – d) Organotypic slices stained for Ki67 (b – d) and cleaved caspase-3 (b – c) at day 4 after treatment with scalar doses of cisplatin, methotrexate, and doxorubicin.

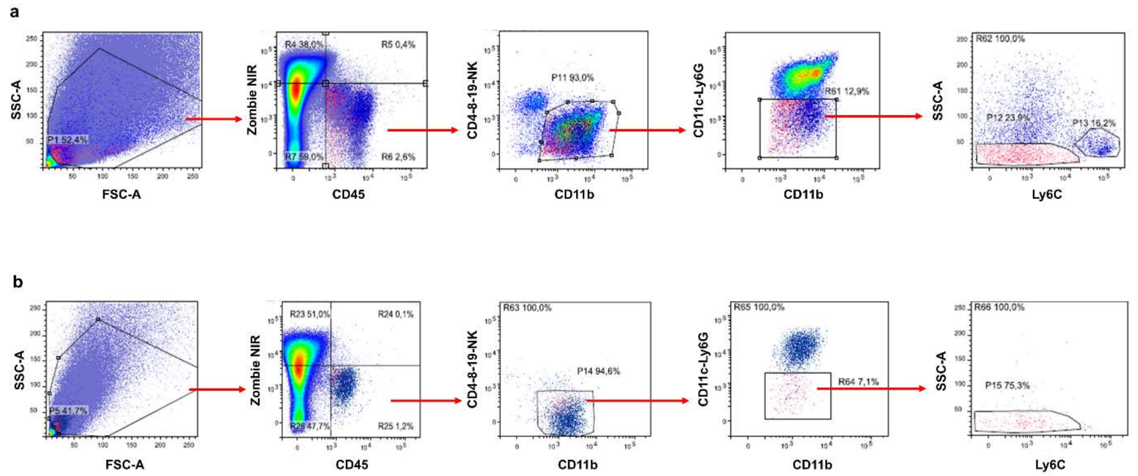

Figure S3. Flow cytometry gating of K-HOS (human osteosarcoma) tumours. Gating strategy for macrophages at (a) day 0 and (b) day 4. For both timepoints, viable immune cells were determined using CD45 and Zombie NIR; T-lymphocytes (CD8+ and CD4+), B lymphocytes (CD19+), natural killer cells, dendritic cells (CD11c+), and neutrophils (Ly6G+) were excluded from the analysis. The macrophage population was identified as positive for CD11b and low/med and high for Ly6C, which defines two subpopulations of macrophages and monocytes.

Supplementary Data

Materials

Human Osteosarcoma Cell line and culture

Human Osteosarcoma cell lines (K-HOS) were used for *in vivo* experiments. K-HOS were purchased from the American Type Culture Collection and were cultured in DMEM high glucose (Lonza) supplemented with 10% FBS (Eurobio) and 1% Penicillin/Streptomycin (Lonza). K-HOS were amplified in 175 cm^2 tissue culture flasks (Corning) at 37°C in a humidified tissue culture incubator with 5% CO^2. After 3 or 4 days, cells were detached using Trypsin-EDTA (Eurobio), resuspended in DMEM supplemented with 10% FBS, centrifuged and washed in PBS (Lonza). Cells were counted and resuspended in PBS before injection into para tibial muscles of Nude NMRI mice.

Human model of osteosarcoma

Four-week-old NMRI-nude mice from Janvier Labs were housed under pathogen-free conditions in the animal facilities at the Faculty of Medicine of Nantes. The human osteosarcoma model was approved by the Regional Ethics Committee on Animal Experimentation (CEEA 6) and the Ministry of Research (APAFIS#8405-2017010409498904 v3). Mice were anesthetized before receiving intra-muscular para-tibial injection of 2 x 10^6 K-HOS cells in 50 μL of PBS. Mice were observed for tumour growth twice per week until the tumour reached the approximate volume of 1000 mm^3.

Methods

Flow cytometry on HOS organotypic slices

Multicolour flow cytometry was used to study the tumour microenvironment associated with 3D organotypic cultures at days 0 and 4 after vibratome slicing. As with the murine osteosarcoma model, dissociation was carried out with gentle MACS dissociator (Miltenyi Biotec). To monitor the microenvironment of organotypic explant slices, a fluorescent conjugated antibody cocktail was set up: CD45 BV650 (BD Biosciences), CD11b AF700 (BD Biosciences), Ly6C PeCy7 (Biolegend), CD4-PercpCy5.5 (BD Biosciences), CD8a-PercpCy5.5 (BD Biosciences), CD19 PercpCy5.5 (BD Biosciences), NK1.1 PercpCy5.5 (BD Biosciences), CD11C-PE (BD Biosciences), Ly6G-PE (BD Biosciences). Zombie NIR was used to exclude dead cell from analysis. Compensations were set up

using Versacomp Antibody Capture beads (Beckman coulter) and all acquisitions were done using BD LSR Fortessa X-20. All data were analysed with FlowLogic 7.2.1 Software (Inivai technologies, Miltenyi Biotec).

Results

The tumour microenvironment was monitored on 3D organotypic culture at days 0 and 4 after vibratome slicing. Flow cytometry gating strategy was designed to focus on monocytes and macrophages. CD4, CD8, CD19 and NK (Natural Killer) cells were eliminated from analysis using the same fluorochrome. Then, dendritic cells (CD11c) and neutrophils (Ly6G) were eliminated, and a gating was done on CD11b cells. The last gate shows monocytes and macrophages populations using Ly6C marker versus SSC-A. At day 0, the two subsets Ly6C low-med and Ly6C high are visible, but total events are low (1330 events for macrophages). At day 4, only Ly6C low-med is visible and the number of events is very low for macrophages: 213 events, which was not sufficient to perform further analysis.
